# Perceived digital health information support and self-rated health among Chinese adults: physical activity mediation and chronic health condition moderation

**DOI:** 10.3389/fpubh.2026.1919116

**Published:** 2026-09-03

**Authors:** Xiaochu Yang, Xiang Fu, Zexin Wu, Linying Xiao, Ming Luo

**Affiliations:** 1 School of Physical Education and Health, Guangdong Polytechnic Normal University, Guangzhou, China; 2 School of Physical Education, Hunan University of Technology, Zhuzhou, China

**Keywords:** chronic health conditions, digital health exclusion, digital health literacy, health equity, perceived digital health information support, physical activity, self-rated health

## Abstract

**Background:**

Digital health resources provide new opportunities for health information seeking, decision-making, and self-management. Yet residents differ in how they understand, evaluate, and use online health information. This study focuses on perceived digital health information support, namely the perceived usefulness of internet-based information for medical understanding and health-related decision support. Evidence remains limited on how this perceived support relates to self-rated health through physical activity and whether this association differs by chronic health needs and population characteristics.

**Objective:**

This study examined the association between perceived digital health information support and self-rated health from a health equity perspective, tested the mediating role of physical activity, and examined moderation by chronic health conditions and subgroup-specific patterns.

**Methods:**

This cross-sectional study used data from the Chinese General Social Survey 2021. The analytic sample consisted of 2,690 respondents from the CGSS 2021 health module. Statistical analyses were performed using Stata 18.0. Multivariable linear regression used community-clustered robust standard errors and province fixed effects. Bootstrap mediation analysis used 5,000 resamples. A second-stage moderated mediation model tested chronic health conditions. Weighted models, complete-case sensitivity analyses, formal bootstrap contrasts for subgroup differences, and ordered logit models including the physical activity by chronic condition interaction were used as robustness checks.

**Results:**

Perceived digital health information support was positively associated with self-rated health and physical activity. Physical activity represented a statistically significant indirect component of the observed association between perceived support and self-rated health; this indirect component accounted for 9.35% of the observed total association. Chronic health conditions strengthened the observed association between physical activity and self-rated health. Formal bootstrap contrasts indicated that the indirect association differed significantly by age, whereas contrasts by sex, marital status, and socioeconomic status were not statistically significant.

**Conclusions:**

Perceived digital health information support was associated with self-rated health among Chinese adults. Physical activity was one behavioral pathway statistically linking perceived online health information support to health evaluation, especially among adults with continuing health needs. Digital health interventions should combine accessible information support with feasible physical activity opportunities and offline behavioral support to avoid widening digital health inequities.

## 1 Introduction

The internet, mobile health applications, and online health services have become part of daily life. They are changing how residents obtain health knowledge, understand medical advice, and manage their health (1, 2). Digital health resources make health information search, disease prevention, and lifestyle management more convenient. They also create opportunities to improve public health service access and digital health management (3, 4). Yet the expansion of digital health resources does not mean that all residents benefit equally. Online health information often comes from complex sources, its quality varies, and some recommendations may conflict (5, 6). Against this background, the present study focuses on perceived digital health information support: the extent to which residents perceive internet-based health information as useful for understanding medical advice, judging whether care is needed, and confirming medical recommendations. This focus is narrower than comprehensive digital health literacy, but it is important for digital health equity because health information is more likely to matter when it is perceived as understandable, trustworthy, and usable in everyday health decisions (7, 8).

Many studies have shown that digital health literacy is closely related to health outcomes and health behaviors. Higher digital health literacy is associated with better health knowledge, stronger disease prevention awareness, chronic disease self-management, and better self-rated health (9–11). It is also associated with health behaviors such as physical activity, dietary management, and preventive health actions (10, 12). However, digital health information does not automatically become health improvement. Online health information can include misinformation, commercial content, and conflicting suggestions, which may increase the difficulty of judging its relevance and quality (5, 6). This distinction is important because perceived support from digital health information may be beneficial only when it is connected with actual behavioral practices and accessible health resources.

Researchers have begun to examine mechanisms linking digital health literacy-related constructs to health. Prior studies often explain this relationship through health management capacity, self-efficacy, health decision confidence, chronic disease self-management, and patient-provider communication (1, 9, 13). Less attention has been paid to behavioral practice in everyday life. Physical activity is an important behavior for health promotion and chronic disease prevention (10, 12, 14). Compared with more specialized behaviors such as medication adherence or clinical service use, physical activity is a daily, modifiable, and widely recommended behavior that can connect health information with bodily experience, function, and self-rated health. Chronic health conditions may also affect this association because people with chronic disease, long-term illness, or disability may evaluate health changes against a different functional and symptom-related baseline (15, 16).

Digital health information support and physical activity may also vary by sex, age, marital status, and socioeconomic status. Digital divides and socioeconomic resources affect the capacity to obtain, understand, and use digital health information. They may also contribute to unequal health behaviors and health outcomes (17–20). Digital inequality is not limited to internet access; it also includes differences in information interpretation, resource conversion, and the availability of offline support that allows digital information to become meaningful health action (19–23). This makes it necessary to examine group-specific patterns in the association among perceived digital health information support, physical activity, and self-rated health.

This study examined the association between perceived digital health information support and self-rated health. It focused on the mediating role of physical activity, tested whether chronic health conditions moderated the association between physical activity and self-rated health, and compared subgroup-specific indirect associations across sex, age, marital status, and socioeconomic status. Because all individual-level variables were measured at the same time, the findings are interpreted as statistical associations and conditional indirect associations rather than evidence of temporal or causal mechanisms.

## 2 Theoretical framework and hypotheses

### 2.1 Theoretical analysis framework

The integrated model of health literacy emphasizes the movement from health information to health decision-making and action. Health information can support health decisions only when individuals are able to access, understand, appraise, and apply it (7, 24). This sequence is particularly relevant in digital health environments, where residents encounter health information from online platforms, teleconsultation, social media, and health applications, and where the credibility, readability, and applicability of information may vary (1, 5, 8). Perceived digital health information support is therefore understood as residents' perceived usefulness of internet-based information for making sense of medical advice and care-related choices. It captures a subjective information-support condition that may shape whether online health information becomes meaningful for everyday health management.

The Health Belief Model extends this information-processing logic to behavioral decision-making. The model emphasizes perceived risk, perceived benefits, barriers, cues to action, and self-efficacy as determinants of health behavior (25, 26). In digital health settings, online information may operate as a cue to action when it helps residents recognize the relevance of physical activity, evaluate possible benefits and risks, and judge whether an activity plan is feasible (27–30). Thus, the theoretical link in this study runs from perceived online information support to health-related judgment and then to behavior. Perceived digital health information support is expected to be associated with self-rated health partly when this perceived support is connected with actual physical activity participation, rather than when online information remains only a general source of exposure.

Digital health literacy is broader in theory and includes information search, evaluation, application, communication, privacy protection, and device operation (1, 8). Accordingly, the present study does not treat the three health-module items as an objective or complete test of digital health literacy. Instead, the measured construct is interpreted as perceived online health information support, especially support for understanding doctors, judging whether care is needed, and confirming medical advice. The following hypotheses therefore concern perceived support from online health information rather than objective digital health literacy skills.

### 2.2 Perceived digital health information support and self-rated health

Perceived digital health information support refers to residents‘ perceived support from internet-based health information in understanding, judging, and applying health-related advice. It differs from objective digital health literacy because it does not directly test respondents' actual skills. Nevertheless, perceived support may still matter for health evaluation because it can reduce uncertainty when residents interpret medical advice and health information. Self-rated health is a broad health measure that reflects overall judgments about physical condition, functional status, and general health, and it predicts later health changes (31–33). Previous research has linked digital health information search and evaluation with physical health, mental health, life satisfaction, and health behaviors (4).

**Hypothesis 1:** Perceived digital health information support is positively associated with self-rated health.

### 2.3 Mediating role of physical activity

Physical activity is an important health-promoting behavior. It can improve physical function, metabolic health, and mental health, and it is linked to lower chronic disease risk and premature mortality (34–36). Prior research also shows that eHealth literacy and digital health literacy are associated with exercise participation and physical activity levels (12, 37). Among multiple possible health behaviors, physical activity is theoretically appropriate for this study because it is both information-sensitive and experience-based. It can be influenced by health advice, risk appraisal, perceived benefits, and feasibility judgments, while its bodily consequences may be directly reflected in energy, symptoms, functional capacity, and self-rated health (25–30, 34–38). For this reason, physical activity provides a plausible behavioral bridge between perceived online information support and overall health evaluation.

From a behavioral decision perspective, residents who perceive internet-based health information as useful may be more able to understand exercise advice, judge whether it is appropriate for them, and plan feasible activity. This does not imply that online information automatically produces behavior change. Rather, physical activity is treated as a statistical behavioral pathway consistent with the proposed theoretical logic: perceived information support may lower uncertainty and strengthen action cues, while physical activity provides the concrete practice through which such support may become health-relevant (10, 12, 25–28, 37).

**Hypothesis 2:** Physical activity participation mediates the association between perceived digital health information support and self-rated health.

### 2.4 Moderating role of chronic health conditions

Self-rated health is a comprehensive subjective evaluation. People form this judgment by considering function, disease burden, and daily experience (31, 32). Adults with chronic disease, long-term illness, or disability may be more sensitive to health-related changes because persistent symptoms and functional limitations provide a salient reference for evaluating health (16, 39). In this context, differences in physical activity frequency may be more strongly associated with self-rated health because physical activity can be linked to functional maintenance, energy, symptom experience, and self-management.

This study therefore interprets chronic health status as a moderator of the observed association between physical activity and self-rated health, not as evidence that physical activity produces larger causal returns among people with chronic conditions. Adults without chronic health problems may have a different baseline health distribution and may evaluate physical activity gains less immediately. These factors suggest that physical activity may show a stronger observed association with self-rated health among adults with chronic health problems.

**Hypothesis 3:** Chronic health conditions positively moderate the association between physical activity participation and self-rated health. This association is stronger among adults with chronic disease, long-term illness, or disability.

### 2.5 Differences across population groups

The health relevance of digital health resources may not be equal across population groups. Age, sex, marital status, and socioeconomic status may affect digital health information access and digital resource use. They may also shape health behavior practice and health outcomes (17, 18, 20). Following standard epidemiological practice, education and income were used as indicators of socioeconomic position (40). Older adults and people with chronic health needs may face greater health management pressure. Marital relationships may provide reminders, emotional support, and shared activity opportunities. These factors may shape subgroup-specific patterns in the statistical indirect association.

**Figure 1:**
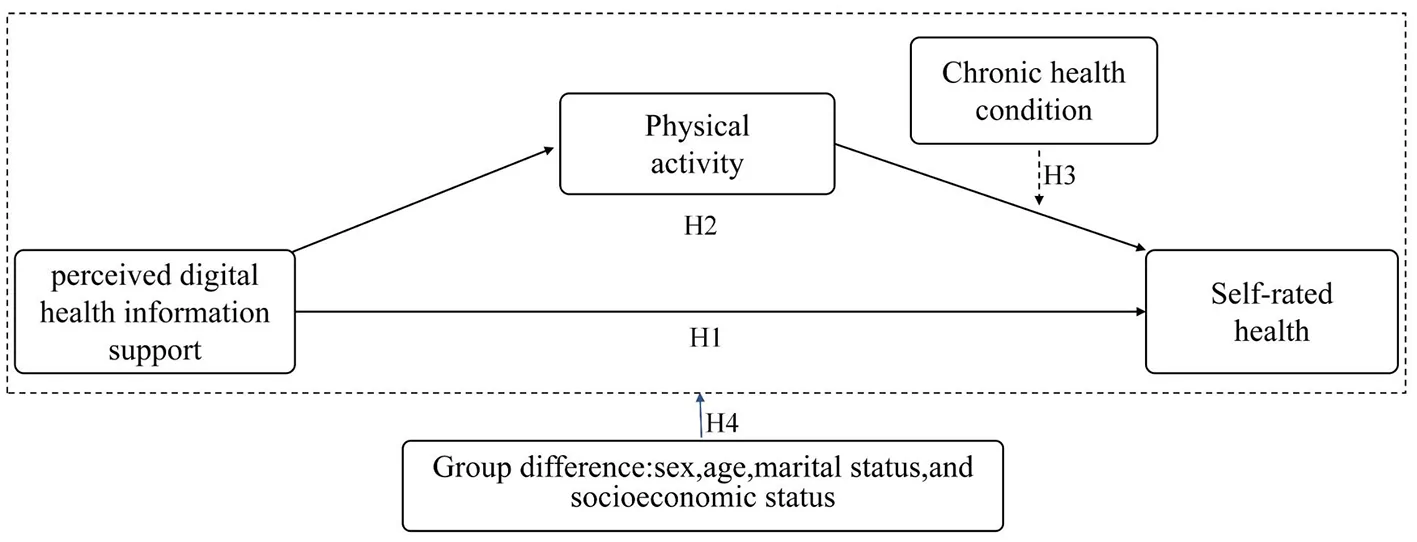
Hypothesized model.

**Hypothesis 4:** The indirect association from perceived digital health information support to self-rated health through physical activity differs across population groups. This hypothesis is evaluated using subgroup-specific indirect effects and formal bootstrap contrasts. The hypothesized model of this study is presented in Figure 1.

## 3 Materials and methods

### 3.1 Study design and data source

This study was a cross-sectional study based on secondary data from the Chinese General Social Survey 2021. CGSS is a large-scale, continuing social survey in China that collects information on demographic characteristics, socioeconomic status, internet use, health information, health behaviors, and health status. The health-related variables used in this study were drawn from the CGSS 2021 health module. According to the CGSS 2021 survey design, all respondents completed the core module and topic module, whereas the additional EASS health module, ISSP health module, and ISSP environment module were each administered to a randomly selected one-third subsample (41).

**Figure 2:**
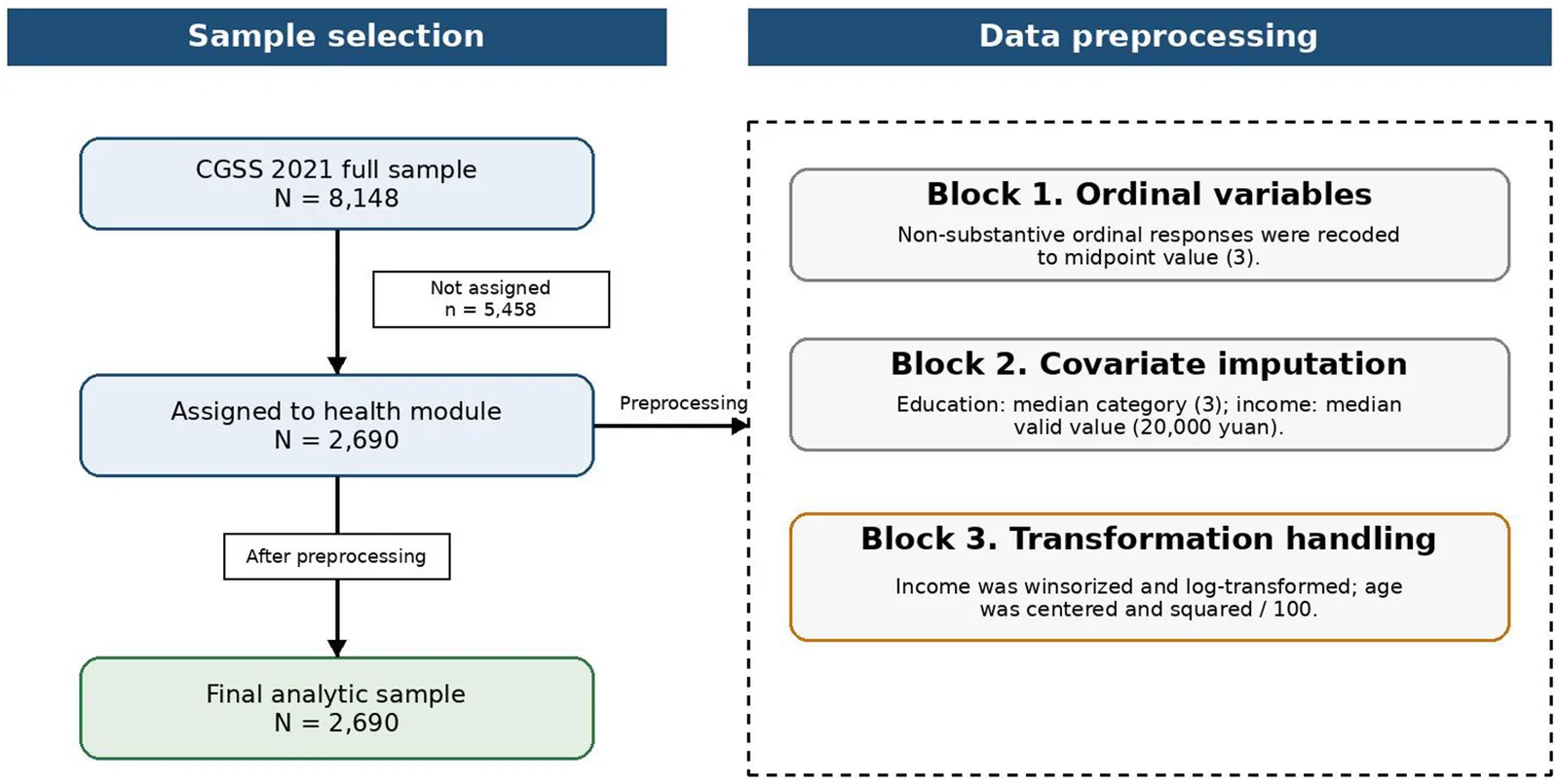
Sample selection and data preprocessing flowchart.

The original CGSS 2021 dataset included 8,148 respondents. A total of 2,690 respondents were assigned to the health module containing the study variables and were retained as the primary analytic sample. The remaining 5,458 respondents were not treated as complete-case exclusions; they were not assigned to the relevant health module. These sample-selection and preprocessing steps are shown in Figure 2.

The decision to retain the full health-module sample was based on two considerations. First, the module respondents were generated by the CGSS modular sampling design rather than by researcher-defined convenience sampling. Second, retaining the health-module sample avoided unnecessary loss of observations from additional listwise deletion. Missing-data methodology recognizes that complete-case deletion can reduce precision and may introduce bias when missingness is related to observed variables (42, 43). Therefore, transparent recoding and imputation were used for limited non-substantive or missing responses, and alternative handling strategies were examined in sensitivity analyses.

Before model estimation, data processing was conducted for ordinal responses and covariates. For ordinal study variables with a substantive midpoint, non-substantive responses such as “do not know,” “unable to choose,” and “refused to answer” were recoded to the midpoint value before reverse coding or scale construction. For covariates, missing education values were imputed using the median category. Special or missing personal annual income responses were set to missing and imputed using the median valid income value of 20,000 yuan; 251 income observations (9.33%) were imputed. Income was then winsorized at the 1st and 99th percentiles and transformed as ln (income + 1). Age was mean-centered before entering regression models, and the quadratic age term was calculated as centered age squared divided by 100. Median imputation was used as a transparent single-imputation strategy for control variables with limited missingness, while sensitivity analyses were conducted to examine whether the main findings depended on this choice (42, 43).

### 3.2 Measures

The outcome variable was self-rated health. It was measured by the question, “In general, how would you rate your health?” Responses were reverse-coded from 1 to 5, with higher scores indicating better self-rated health. Self-rated health is a single-item measure that reflects disease status, physical function, psychological feelings, and life experience and is widely used in population health research (31–33, 44).

The independent variable was perceived digital health information support. It was constructed from three CGSS 2021 items assessing whether internet-based information helps respondents understand doctors, judge whether medical care is needed, and confirm whether medical advice is appropriate. The original wording of these items does not directly ask respondents to report objective skills or confidence in performing all digital health literacy tasks. Therefore, the construct is interpreted as perceived support from online health information rather than a complete measure of digital health literacy. The three items were reverse-coded and averaged so that higher scores indicated stronger perceived support. Cronbach's alpha was 0.682. The KMO value was 0.621, Bartlett's test was significant, one common factor explained 61.492% of the variance, and the factor loadings were 0.671, 0.827, and 0.843.

The mediator was physical activity participation. It was measured by the frequency of exercise lasting at least 20 minutes and causing sweating or faster breathing. Responses were coded from 1 to 5, with 1 indicating never, 2 once a month or less, 3 several times a month, 4 several times a week, and 5 daily. Higher scores indicated more frequent physical activity participation in this measured exercise category.

The moderator was chronic health condition. It was measured by whether the respondent had long-term illness, chronic disease, or disability. The original item did not distinguish disease type, severity, or functional limitation. Responses were coded as 0 = no and 1 = yes; non-substantive responses were imputed using the modal category to preserve the health-module sample.

Covariates included sex, age, age squared, marital status, education, personal income, and urban residence. Province fixed effects were also included. Sex was coded as male = 0 and female = 1. Marital status was coded as cohabiting or partnered = 1 and other = 0. Education was coded from 1 to 5. The categories were no formal education, primary school, junior high school, senior high or vocational education, and college or above. Urban residence was coded as rural = 0 and urban = 1.The measurement items and coding schemes for all study variables are summarized in Table 1.

**Table 1:** Variable definitions and measurements.

Variable	Measurement items	Assignment instructions
Self-rated health	In general, how would you rate your health?	Reverse-coded as: Very poor = 1; Poor = 2; Fair = 3; Good = 4; Very good = 5.
Perceived digital health information support: information understanding	Internet information helps me understand what doctors tell me.	Reverse-coded as: Strongly disagree = 1; Disagree = 2; Neither agree nor disagree = 3; Agree = 4; Strongly agree = 5.
Perceived digital health information support: information evaluation	The internet helps people judge whether they need to see a doctor.	Reverse-coded as: Strongly disagree = 1; Disagree = 2; Neither agree nor disagree = 3; Agree = 4; Strongly agree = 5.
Perceived digital health information support: information application	The internet helps confirm whether doctors have given appropriate advice.	Reverse-coded as: Strongly disagree = 1; Disagree = 2; Neither agree nor disagree = 3; Agree = 4; Strongly agree = 5.
Physical activity participation	How often do you engage in physical exercise lasting at least 20 minutes and causing sweating or faster breathing?	Never = 1; Once a month or less = 2; Several times a month = 3; Several times a week = 4; Daily = 5.
Education	Highest educational attainment	No formal education = 1; Primary school = 2; Junior high school = 3; Senior high school or secondary vocational education = 4; College or above = 5.
Chronic health condition	Whether the respondent has long-term illness, chronic disease, or disability.	No = 0; Yes = 1.
Sex	Respondent sex	Male = 0; Female = 1.
Age	Respondent age	Continuous variable; age was mean-centered. Age squared refers to centered age squared/100.
Marital status	Current marital status	Cohabiting or partnered = 1; Other marital statuses = 0.
Personal income	Personal annual income	Special or missing values were imputed using the median valid income value, winsorized at the 1st and 99th percentiles, and transformed as ln(income + 1).
Urban residence	Residence type	Rural = 0; Urban = 1.

### 3.3 Statistical analysis

Descriptive statistics summarized the main variables. Both unweighted and weighted descriptive statistics were examined. Spearman correlations assessed initial associations among core variables. Harman's single-factor test was used as a diagnostic check for common method bias. Variance inflation factors assessed multicollinearity.

Multivariable linear regression tested associations among perceived digital health information support, physical activity, and self-rated health. OLS was retained as the main estimator because it allows direct decomposition of the indirect association and straightforward interpretation of mediation and moderated mediation coefficients. Because self-rated health and physical activity are ordered variables, ordered logit models were used as sensitivity analyses. Bootstrap mediation analysis used 5,000 resamples to test the indirect effect (45). A second-stage moderated mediation model then tested chronic health conditions (46).

Socioeconomic status was constructed as a transparent SES index. Education and log-transformed personal income were standardized and averaged, and respondents were divided into higher- and lower-SES groups according to the median of the index. This approach follows the common use of education and income as indicators of socioeconomic position in health research (40). Multigroup mediation analysis compared pathways by sex, age, marital status, and socioeconomic status. Formal bootstrap contrasts were used to compare subgroup-specific indirect effects.

All regression models used community-clustered robust standard errors and controlled for major covariates and province fixed effects. Grouping variables were not repeated in their corresponding subgroup models. The cleaned health-module sample included 320 communities and 19 provinces. Analyses were conducted using Stata 18.0 (StataCorp LLC, College Station, TX, USA). Stata was used to implement regression models, community-clustered robust standard errors, weighted analyses, ordered logit models, marginal predictions, and bootstrap resampling at the community level. Sensitivity analyses included models without province fixed effects, weighted models using the available CGSS sampling weight, a complete-case analysis that treated non-substantive responses in core variables as missing, ordered logit models, and an ordered logit interaction model for physical activity by chronic health condition. Because the data were cross-sectional, temporal order could not be identified. Therefore, all findings indicate statistical associations and conditional indirect associations and should not be interpreted as causal effects.

## 4 Results

### 4.1 Common method bias and multicollinearity

Core variables were self-reported. Harman's single-factor test extracted two factors with eigenvalues above one. The first factor explained 29.044% of the variance. This result suggested that common method bias was not likely to be dominated by a single common factor and, to some extent, reduced concern about common method bias (47). Multicollinearity was also assessed. VIF values ranged from 1.032 to 1.692, and tolerance values ranged from 0.591 to 0.969, indicating no serious multicollinearity (48).

### 4.2 Descriptive statistics and correlations

Detailed category distributions for the ordinal study variables are reported in Table 2. The weighted means for perceived digital health information support, physical activity, and self-rated health were 3.284, 2.866, and 3.005, respectively. Weighted proportions were 50.3% for women, 64.8% for urban residence, 73.7% for partnered respondents, and 27.6% for chronic health conditions. Table 3 reports descriptive statistics and Spearman correlations. The analytic sample included 2,690 health-module respondents. The mean perceived digital health information support score was 3.255. The mean physical activity score was 2.843. The mean self-rated health score was 2.912. Women accounted for 55.7% of the sample, urban residents for 56.9%, partnered respondents for 73.5%, and respondents with chronic health conditions for 34.8%. The core variables were positively correlated. Perceived digital health information support was positively correlated with physical activity and self-rated health, and physical activity was positively correlated with self-rated health. Age was negatively correlated with perceived support, physical activity, and self-rated health.

**Table 2:** Category distributions of ordinal study variables.

Variable	Category 1	Category 2	Category 3	Category 4	Category 5
Self-rated health	249 (9.3%)	951 (35.4%)	531 (19.7%)	707 (26.3%)	252 (9.4%)
Information understanding	68 (2.5%)	255 (9.5%)	1,259 (46.8%)	1,015 (37.7%)	93 (3.5%)
Information evaluation	65 (2.4%)	540 (20.1%)	1,045 (38.8%)	961 (35.7%)	79 (2.9%)
Information application	39 (1.4%)	355 (13.2%)	1,145 (42.6%)	1,074 (39.9%)	77 (2.9%)
Physical activity	867 (32.2%)	392 (14.6%)	371 (13.8%)	417 (15.5%)	643 (23.9%)

**Table 3:** Descriptive statistics and spearman correlations.

Variable	*M*	SD	1	2	3	4	5	6
1. Perceived digital health information support	3.255	0.636	1					
2. Physical activity participation	2.843	1.588	0.084^***^	1				
3. Self-rated health	2.912	1.164	0.100^***^	0.132^***^	1			
4. Education	3.179	1.272	0.145^***^	0.185^***^	0.231^***^	1		
5. Log income	8.227	4.086	0.099^***^	0.099^***^	0.170^***^	0.414^***^	1	
6. Age	51.297	17.460	−0.174^***^	−0.100^***^	−0.263^***^	−0.515^***^	−0.194^***^	1

*N* = 2,690. *M* indicates mean. SD indicates standard deviation. ^***^p < 0.001.

### 4.3 Mediation analysis

Table 4 presents the multivariable regression results. Perceived digital health information support was positively associated with self-rated health and physical activity after adjustment for covariates, province fixed effects, and community-clustered robust standard errors. When physical activity was added to the self-rated health model, physical activity was positively associated with self-rated health and the coefficient for perceived support decreased but remained significant.

**Table 4:** Regression results for perceived digital health information support, physical activity, and self-rated health.

Variable	Model 1 self-rated health	Model 2 physical activity	Model 3 self-rated health
Perceived digital health information support	0.090^***^ (0.034)	0.149^***^ (0.048)	0.081^**^ (0.034)
Physical activity participation	–	–	0.056^***^ (0.013)
Female	−0.037 (0.041)	−0.227^***^ (0.063)	−0.024 (0.040)
Age	−0.003^*^ (0.001)	−0.002 (0.002)	−0.003^*^ (0.001)
Age squared	0.033^***^ (0.008)	−0.022^**^ (0.011)	0.035^***^ (0.007)
Partnered	0.034 (0.053)	0.129 (0.084)	0.027 (0.052)
Education	0.044^**^ (0.021)	0.176^***^ (0.031)	0.034 (0.022)
Log income	0.011^**^ (0.005)	0.003 (0.008)	0.010^**^ (0.005)
Urban residence	0.022 (0.047)	0.132^*^ (0.077)	0.014 (0.047)
Chronic health condition	−0.979^***^ (0.049)	−0.046 (0.080)	−0.977^***^ (0.049)
Province fixed effects	Yes	Yes	Yes
*N*	2,690	2,690	2,690
*R* ^2^	0.248	0.058	0.254

Unstandardized coefficients are reported. Community-clustered robust standard errors are shown in parentheses. Age was mean-centered; age squared refers to centered age squared divided by 100. ^*^p < 0.05; ^**^p < 0.01; ^***^p < 0.001.

Table 5 reports the bootstrap mediation results. The indirect effect through physical activity was 0.0084, and both the percentile 95% CI [0.0024, 0.0156] and bias-corrected 95% CI [0.0030, 0.0165] excluded zero. The indirect component through physical activity accounted for 9.35% of the observed total association. These results supported H2 as a statistical mediation result, not as evidence of a temporal causal mechanism.

**Table 5:** Bootstrap test of the mediation effect of physical activity.

Effect	Estimate	Bootstrap SE	Percentile 95% CI	Bias-corrected 95% CI	Relative component (%)
a path: support to physical activity	0.1486	0.0483	[0.0517, 0.2437]	[0.0547, 0.2458]	/
b path: physical activity to self-rated health	0.0565	0.0132	[0.0303, 0.0818]	[0.0309, 0.0823]	/
Indirect effect a^*^b	0.0084	0.0034	[0.0024, 0.0156]	[0.0030, 0.0165]	9.35
Direct effect c'	0.0814	0.0338	[0.0164, 0.1478]	[0.0135, 0.1448]	90.65
Total effect c	0.0898	0.0339	[0.0249, 0.1565]	[0.0220, 0.1536]	100.00

Bootstrap resampling was performed at the community level with 5,000 resamples. Relative component means the share of the observed total association represented by each component.

### 4.4 Moderated mediation by chronic health conditions

Table 6 reports the moderated mediation results testing whether the observed association between physical activity and self-rated health differed by chronic health condition. The self-rated health equation included the interaction term. The interaction between physical activity and chronic health condition was positive and significant (*B* = 0.0822, 95% *CI* [0.0302, 0.1302]), supporting H3. Among adults without chronic health problems, the simple slope was weak and not significant (*B* = 0.0236, 95% *CI* [-0.0092, 0.0579]). Among adults with chronic disease, long-term illness, or disability, the estimated slope was 0.1058.

**Table 6:** Moderated mediation by chronic health conditions.

Effect/path	Estimate	Bootstrap SE	95% CI
a path: support to physical activity	0.1486	0.0483	[0.0517, 0.2437]
b path among adults without chronic health problems	0.0236	0.0171	[-0.0092, 0.0579]
Physical activity x chronic health condition	0.0822	0.0253	[0.0302, 0.1302]
Conditional indirect effect without chronic health problems	0.0035	0.0028	[-0.0014, 0.0098]
Conditional indirect effect with chronic health problems	0.0157	0.0061	[0.0049, 0.0288]
Index of moderated mediation	0.0122	0.0059	[0.0025, 0.0257]
Direct effect c'	0.0835	0.0337	[0.0192, 0.1501]

The model is equivalent to a second-stage moderated mediation model. Chronic health condition was coded 0 = no and 1 = yes.

Conditional indirect effects also supported moderated mediation. Among adults without chronic health problems, the indirect effect was 0.0035 and its 95% CI included zero. Among adults with chronic health problems, the indirect effect was 0.0157 and its 95% *CI* excluded zero. The index of moderated mediation was 0.0122, with a 95% *CI* of [0.0025, 0.0257]. An ordered logit interaction model provided consistent support for this moderation pattern; the physical activity by chronic health condition interaction was significant (*OR* = 1.248, 95% *CI* [1.136, 1.372], *p* < 0.001). Predicted probabilities from the ordered logit interaction model are reported in Table 7.

**Table 7:** Predicted category probabilities from the ordered logit interaction model.

Chronic	PA	Very poor	Poor	Fair	Good	Very good
No	1	0.034 [0.028, 0.043]	0.291 [0.265, 0.323]	0.232 [0.213, 0.249]	0.325 [0.299, 0.347]	0.118 [0.099, 0.139]
	2	0.033 [0.028, 0.040]	0.285 [0.265, 0.308]	0.231 [0.213, 0.247]	0.330 [0.307, 0.349]	0.121 [0.106, 0.139]
	3	0.032 [0.028, 0.039]	0.279 [0.260, 0.299]	0.230 [0.212, 0.245]	0.334 [0.312, 0.353]	0.125 [0.110, 0.140]
	4	0.031 [0.026, 0.038]	0.273 [0.251, 0.296]	0.229 [0.211, 0.245]	0.339 [0.314, 0.359]	0.128 [0.113, 0.146]
	5	0.030 [0.025, 0.038]	0.267 [0.236, 0.297]	0.227 [0.210, 0.245]	0.343 [0.313, 0.370]	0.132 [0.113, 0.154]
Yes	1	0.256 [0.224, 0.292]	0.559 [0.529, 0.579]	0.106 [0.092, 0.123]	0.065 [0.054, 0.080]	0.013 [0.011, 0.017]
	2	0.212 [0.191, 0.240]	0.563 [0.535, 0.585]	0.126 [0.112, 0.141]	0.082 [0.070, 0.096]	0.017 [0.014, 0.022]
	3	0.173 [0.155, 0.197]	0.556 [0.525, 0.579]	0.147 [0.132, 0.162]	0.102 [0.088, 0.116]	0.022 [0.019, 0.027]
	4	0.140 [0.123, 0.166]	0.537 [0.507, 0.562]	0.169 [0.152, 0.186]	0.125 [0.108, 0.142]	0.028 [0.024, 0.035]
	5	0.113 [0.093, 0.140]	0.510 [0.474, 0.540]	0.189 [0.169, 0.210]	0.152 [0.129, 0.177]	0.036 [0.029, 0.046]

Cells show predicted probability [95% CI]. PA, physical activity level.

Physical activity levels range from 1 = never to 5 = daily.

Predictions are marginal standardized probabilities from the ordered logit model with covariates and province fixed effects.

### 4.5 Multigroup mediation analysis

Table 8 reports subgroup-specific indirect effects and formal bootstrap contrasts. Socioeconomic status was measured using an index based on education and personal income, two commonly used indicators of socioeconomic position in health research. Education and log-transformed personal income were standardized and averaged to form the SES index. Respondents were then divided into higher- and lower-SES groups according to the median value of the index. The indirect effect was significant among men but not women; however, the sex contrast was not statistically significant. The indirect effect was significant in the lower-SES group and marginally similar in the higher-SES group, but the SES contrast was not significant. The age comparison showed a clear difference: the indirect effect was significant and larger among older adults, whereas it was not significant among non-older adults, and the bootstrap contrast excluded zero. The indirect effect was significant among partnered respondents but not among unpartnered respondents; however, the marital-status contrast was not significant.

**Table 8:** Subgroup-specific indirect effects and formal bootstrap contrasts.

Comparison	Group	*N*	Indirect effect	95% bootstrap CI	Contrast in indirect effect	95% bootstrap CI for contrast
Sex	Men	1,191	0.0110	[0.0019, 0.0230]	0.0056	[-0.0065, 0.0201]
	Women	1,499	0.0054	[-0.0026, 0.0146]		
SES	Higher SES	1,281	0.0063	[-0.0002, 0.0150]	−0.0050	[-0.0187, 0.0081]
	Lower SES	1,409	0.0113	[0.0009, 0.0239]		
Age	Older adults	938	0.0285	[0.0089, 0.0494]	0.0262	[0.0060, 0.0470]
	Non-older adults	1,752	0.0023	[-0.0011, 0.0082]		
Marital status	Partnered	1,977	0.0093	[0.0015, 0.0177]	0.0033	[-0.0099, 0.0157]
	Unpartnered	713	0.0060	[-0.0029, 0.0184]		

Indirect effects represent the pathway through physical activity. Contrasts are calculated as the first subgroup minus the second subgroup. Statistical significance is determined by 95% bootstrap CIs excluding zero.

Therefore, H4 was only partially supported. Formal contrast evidence supported an age difference, while sex, SES, and marital-status results should be interpreted as exploratory subgroup-specific patterns rather than confirmed group differences.

### 4.6 Robustness and sensitivity checks

Table 9 reports robustness and sensitivity checks. Ordered logit models supported the positive associations among perceived support, physical activity, and self-rated health. The ordered logit interaction model also supported the physical activity by chronic condition interaction. Weighted sensitivity models showed that perceived support remained positively associated with physical activity and that physical activity remained positively associated with self-rated health, although the weighted direct association between perceived support and self-rated health was attenuated. Complete-case sensitivity analyses that treated non-substantive responses in core variables as missing produced directions consistent with the main models. Models without province fixed effects also remained directionally consistent.

**Table 9:** Robustness and sensitivity checks.

Check	Indicator	Estimate/OR	95% CI	*p* value
Alternative estimator	Ordered logit: support to physical activity	1.203	[1.074, 1.347]	0.001
	Ordered logit: support to self-rated health	1.150	[1.018, 1.300]	0.025
	Ordered logit: physical activity to self-rated health	1.120	[1.068, 1.175]	< 0.001
Ordered interaction model Weighted model	Physical activity x chronic health condition	1.248	[1.136, 1.372]	< 0.001
	Support to physical activity	0.149	[0.049, 0.250]	0.004
	Physical activity to self-rated health	0.042	[0.015, 0.069]	0.002
	Direct support to self-rated health	0.041	[-0.032, 0.115]	0.273
Complete-case sensitivity	Support to self-rated health	0.087	[0.018, 0.157]	0.014
	Support to physical activity	0.110	[0.005, 0.216]	0.039
	Physical activity to self-rated health	0.051	[0.020, 0.082]	0.001
Alternative model	Without province fixed effects: total association	0.077	[0.011, 0.144]	0.022
	Without province fixed effects: a path	0.151	[0.058, 0.245]	0.002
	Without province fixed effects: direct association	0.068	[0.002, 0.135]	0.044
	Without province fixed effects: b path	0.059	[0.034, 0.084]	< 0.001

Ordered logit models report odds ratios. Linear models report unstandardized coefficients. The complete-case sensitivity analysis retained 1,822 respondents with valid substantive responses on the core ordinal and chronic-condition variables while retaining the same covariate handling strategy. CI indicates confidence interval.

## 5 Discussion

### 5.1 Key findings

This study used CGSS 2021 health-module data to examine perceived digital health information support, physical activity participation, and self-rated health. It also examined chronic health conditions and population characteristics. Perceived digital health information support was positively associated with self-rated health. It was also positively associated with physical activity participation. Physical activity represented a statistically significant but modest indirect component of the observed association between perceived digital health information support and self-rated health. However, the indirect component was small. This suggests that physical activity is one daily behavioral pathway. It is not the only explanation for health evaluation differences. Chronic health conditions strengthened the observed association between physical activity and self-rated health. The conditional indirect association was mainly found among adults with chronic disease, long-term illness, or disability. Formal bootstrap contrasts further showed that the indirect association differed significantly by age, whereas sex, marital-status, and socioeconomic-status patterns should be interpreted as exploratory rather than confirmed group differences.

### 5.2 Perceived digital health information support and self-rated health

The results supported H1. Perceived digital health information support was positively associated with self-rated health. This finding is consistent with prior studies on eHealth literacy and health outcomes (9, 49, 50). Perceived digital health information support differs from general internet use or search frequency. It emphasizes whether residents perceive online health information as helpful for understanding medical advice, judging care-seeking needs, and evaluating medical recommendations. This perceived support is closely related to health decision-making and health management (1, 21). The results suggest that digital health value does not depend only on exposure to online information. It also depends on whether information can be understood, evaluated, and applied to health problems.

This finding has public health implications. Health differences in digital environments may arise from differences in perceived information support and information usability. Residents often face uncertainty about symptoms, medical choices, and medical advice. Residents with stronger perceived digital health information support may better make sense of online information and medical advice. They may form clearer judgments when health problems occur (51, 52). By contrast, residents with limited perceived digital health information support may not gain benefits from digital resources. They may lack sufficient support for judging whether online information is applicable to their own health situation. Digital health service expansion should therefore include not only resource provision but also support for understandable, trustworthy, and usable health information. This is important for reducing differences in digital health resource use and promoting health equity. The interpretation of this result therefore concerns perceived support from online health information rather than objective digital health literacy skills.

### 5.3 Mediating role of physical activity

The mediation results supported H2. Physical activity represented a statistical indirect pathway in the association between perceived digital health information support and self-rated health. This finding suggests that perceived digital health information support can have an indirect association with health evaluation. The indirect association may operate through concrete health behavior. Residents with stronger perceived online health information support may better understand exercise advice and sedentary risk. They may also judge whether exercise information is suitable for them (10, 12, 37). Physical activity is linked to better physical function, health-related quality of life, and self-rated health (36, 38, 53, 54). Therefore, physical activity can be viewed as a behavioral pathway. It may help connect perceived online health information support with health-related evaluation.

The indirect component accounted for 9.35% of the observed total association. This result requires careful interpretation. It supports a statistically significant behavioral pathway linking perceived information support, physical activity, and self-rated health. Yet the explanatory power of this pathway is limited. Digital health information support may relate to self-rated health through other mechanisms. These may include health service use, chronic disease self-management, medication adherence, patient-provider communication, and mental health regulation (51, 52). The physical activity measure also focused on exercise frequency. It did not include daily physical activity, duration, intensity, or activity type. This may underestimate the mediating role of physical activity. Future studies should use more complete physical activity instruments. More broadly, the modest size of the indirect component suggests that online health information support alone cannot substitute for behavioral resources, safe activity opportunities, and offline guidance.

### 5.4 Moderating role of chronic health conditions

The moderated mediation results supported H3. Chronic health conditions significantly moderated the observed association between physical activity and self-rated health. Among adults with chronic disease, long-term illness, or disability, the positive association was stronger. Among adults without chronic health problems, the association was weaker and not significant. This result is consistent with evidence on physical activity and chronic disease management (34–36). For adults with continuing health needs, physical activity may not be only a general lifestyle choice. It may be closely related to daily function, disease management, symptom experience, and health perception (16, 39). This stronger association may reflect greater sensitivity of self-rated health to physical activity differences among adults with chronic health conditions, rather than evidence that physical activity produces larger causal returns in this group. Adults with chronic disease, long-term illness, or disability often have lower baseline health, more salient symptoms, and more frequent self-management experiences; therefore, even modest differences in physical activity may be more visible in their overall health evaluation.

The conditional indirect effects provide further evidence. The statistical pathway from perceived digital health information support to self-rated health through physical activity was significant mainly among adults with chronic health problems. Prior evidence shows that digital health literacy is important for chronic disease management (39). Yet its health relevance often appears through concrete behavior and self-management processes. For people with chronic health problems, stronger perceived digital health information support may help them understand exercise advice. It may also help them recognize health risks. These forms of perceived support may support the connection between online information and physical activity. The predicted probabilities from the ordered logit interaction model further suggest that good or very good self-rated health increased more clearly with physical activity among adults with chronic health conditions than among those without chronic health conditions. These probability differences remain cross-sectional associations and should not be interpreted as causal effects.

### 5.5 Group differences and health equity

Multigroup analysis showed that the pathway was not uniform across residents. It varied by sex, age, marital status, and socioeconomic status. This finding partially supported H4. However, formal bootstrap contrasts showed that only the age difference was statistically supported, whereas sex, marital-status, and socioeconomic-status patterns should be interpreted as exploratory subgroup patterns. Prior studies show that population groups differ in health information access and digital health service use. They also differ in health behavior selection and medical resource use (21, 49). These differences may shape how digital health information support becomes connected with health behavior and health evaluation.

From the socioeconomic perspective, the pathway was observed in both SES groups, but the formal SES contrast was not statistically significant. It was not limited to residents with more resources. Residents with lower socioeconomic status may face constraints in education, income, and living resources. These constraints may limit access to high-quality health services and health promotion resources. However, stronger perceived support from online health information may still support physical activity. It may also be connected with more positive health evaluation. This finding suggests possible future research value for examining whether digital health information support has compensatory significance for low socioeconomic status groups.

From the age perspective, the pathway was stronger among older adults. Older adults often face higher chronic disease risk and stronger health management needs. They may be more likely to connect online health information with health-promoting behaviors. They may also perceive health changes through functional maintenance and risk management (11, 12). From the marital status perspective, partnered residents showed a more stable descriptive pathway. Partner support may help with health information understanding, exercise companionship, and behavior maintenance.

These subgroup patterns extend the health-equity interpretation beyond digital access. Digital inequality operates not only through exposure to online health information, but also through residents' ability to interpret information, judge its relevance, and connect it with feasible health behavior. When socioeconomic resources, family support, community exercise spaces, or professional guidance are unevenly distributed, the practical value of online health information may also become unequal. In this sense, perceived digital health information support is shaped by both digital environments and offline resource conditions. Digital health equity therefore requires attention to the full process from information access and information usability to behavioral opportunity, community resources, and sustained support (19, 20, 22, 23).

Overall, digital health information support may depend on health needs, family support, and socioeconomic resources. Digital health interventions should avoid a one-size-fits-all information-push model. They should provide targeted support based on behavioral support conditions.

### 5.6 Practical implications

This study offers several practical implications for digital health services and health behavior support. First, digital health interventions should include behavioral conversion support. They should not stop at information provision. The mediation component of physical activity was significant but limited. This suggests that digital health information support alone may be insufficient if residents cannot connect information with feasible action. Community health services should provide both information screening and behavior guidance. They could turn online exercise advice into community activity programs. Family doctors and social sports instructors could also provide practical exercise guidance. This may reduce the gap between knowing and doing.

Second, adults with chronic disease, long-term illness, or disability should receive focused support. The moderated mediation results showed that the pathway was mainly significant in this group. Primary care institutions could use family doctor services to provide layered support. Such support could combine online health information screening and offline safe exercise guidance. It could include exercise risk assessment, individualized activity plans, and follow-up. General advice to exercise should not be applied without considering functional limitation or disease risk. For residents with chronic health needs, digital health information support should therefore be linked with chronic-condition-sensitive physical activity guidance rather than delivered as general lifestyle information alone.

Third, digital health interventions should consider group differences. The formally supported age contrast and the exploratory subgroup patterns showed differences by older age, partnership status, and socioeconomic status. Digital health services should not rely on a single information-push approach. They should provide clearer health explanations, physical activity opportunities, and community support. For residents with fewer socioeconomic resources, services should reduce use barriers. Information should be easy to understand and close to daily life. Physical activity guidance should also be accessible and feasible. This may help connect digital health resources with health-supporting behavior and health evaluation.

### 5.7 Limitations and future research

This study has several limitations. First, it used cross-sectional data. The temporal order among perceived digital health information support, physical activity, and self-rated health could not be confirmed. Therefore, causal inference is not possible. Second, perceived digital health information support was constructed from three CGSS 2021 items. These items captured perceived support from online health information for medical understanding, care-seeking judgment, and evaluation of medical advice. They did not capture device operation, platform navigation, active search, cross-checking, or privacy protection. Third, physical activity, self-rated health, and chronic health condition were self-reported. Reporting bias may exist. The physical activity item mainly measured frequency. It could not distinguish duration, intensity, or activity type.

Fourth, the chronic health condition item combined long-term illness, chronic disease, and disability. It could not distinguish disease type, severity, or functional limitation. Fifth, although the health-module respondents were assigned through the CGSS modular design, the analysis remains based on the health-module subsample. Weighted and complete-case sensitivity analyses were conducted, but selection and missing-data concerns cannot be fully eliminated. Finally, the multigroup analysis was exploratory except for the formally supported age contrast. It could not fully explain the mechanisms behind group differences. Future research should use longitudinal data, natural experiments, or digital health information-support interventions. It should also use complete digital health literacy scales, detailed physical activity instruments, disease-specific indicators, and more complete measures of digital health equity.

## 6 Conclusions

This study found a positive association between perceived digital health information support and self-rated health among Chinese adults in the CGSS 2021 health module. Physical activity represented a statistically significant but small indirect component of this observed association. Chronic health conditions strengthened the observed association between physical activity and self-rated health, and ordered logit sensitivity analyses supported this moderation pattern. Formal subgroup contrasts indicated that the indirect association differed by age, while sex, marital-status, and SES patterns were exploratory. Overall, digital health value depends on more than exposure to online information. It also depends on whether information is perceived as supportive, whether residents can translate information into feasible health behavior, and whether offline resources support this process. Digital health interventions should therefore combine information support with accessible physical activity opportunities and chronic-condition-sensitive guidance to promote health equity in digital health environments.

## Data Availability

The data analyzed in this study are derived from the Chinese General Social Survey 2021. The dataset can be accessed through the official CGSS data platform (http://cgss.ruc.edu.cn/). Researchers may apply for access through the CGSS data service system.
